# Ultrasound diagnosis of an unruptured rudimentary horn pregnancy with placenta accreta: a case report

**DOI:** 10.3389/fgwh.2026.1742637

**Published:** 2026-06-17

**Authors:** Guangquan Guo, Jinjin Zhou, Xin Hang, Shuling Cheng, Mengling Li

**Affiliations:** 1 Department of Anesthesiology, Weifang People's Hospital, Shandong, China; 2 Department of Ultrasound, Weifang People's Hospital, Shandong, China; 3 Department of Ultrasound, Hanting District Maternal and Child Health Hospital, Weifang, Shandong, China

**Keywords:** placenta accreta, rudimentary horn pregnancy, surgery, ultrasound, uterine malformation

## Abstract

**Background:**

Rudimentary Horn Pregnancy (RHP) is a rare and potentially life-threatening condition that can closely resemble abdominal pregnancy both clinically and on ultrasound imaging. Therefore, prompt diagnosis is essential to prevent severe complications.

**Case presentation:**

This report describes a case involving a primiparous woman at 20 weeks of gestation. A routine antenatal ultrasound revealed a fetus within a right rudimentary uterine horn with placental implantation. During the early stages of pregnancy, the patient exhibited no symptoms. However, she developed abdominal pain, necessitating an emergency laparotomy. Surgical management included the excision of the right rudimentary horn along with the fallopian tube. The patient's postoperative course was uneventful, and the timely surgical intervention prevented the potential complications of uterine rupture and severe hemorrhage.

**Discussion:**

Early diagnosis of RHP poses a significant challenge, making ultrasound an essential tool for detection and diagnosis. As pregnancies within a rudimentary horn are at high risk for rupture, especially during the second or third trimester, timely diagnosis before rupture is imperative. In this regard, regular ultrasound screening during early pregnancy can improve the likelihood of identifying this condition at an earlier stage, reducing the potential for severe complications.

**Conclusion:**

This case highlights the crucial role of ultrasound in diagnosing RHP and underscores the importance of enhanced clinical awareness. Prompt identification and management are important for minimizing maternal and fetal risks associated with this rare condition.

## Introduction

A rudimentary uterus is a congenital uterine malformation resulting from the incomplete fusion of the paramesonephric ducts during embryonic development. This anomaly typically leads to the formation of a functional unicornuate uterus on one side, while development on the opposite side is arrested, leading to the absence of lower ductal segments and resulting in a rudimentary uterus without a cervical structure. Current literature indicates a concerningly low rate of correct preoperative diagnosis, reported between 22% and 29%, with diagnostic sensitivity further declining as gestation progresses (1). In addition, rudimentary uterus can lead to several complications, such as hematochezia, dysmenorrhea, endometriosis, miscarriage, preterm labor, and ectopic pregnancy (2, 3). It has been found that uterine anomalies affect approximately 5% of women, but pregnancies within a rudimentary uterus are exceptionally rare, with an estimated incidence ranging from 1/160,000 to1/76,000 (4) cases and carry a high risk due to the presence of myometrial dysplasia, which increases the likelihood of rupture. In the event of rupture, severe intra-abdominal hemorrhage may occur and may be life-threatening to the mother. Thus, early diagnosis and intervention are essential to prevent these catastrophic outcomes.

## Case presentation

**Figure 1:**
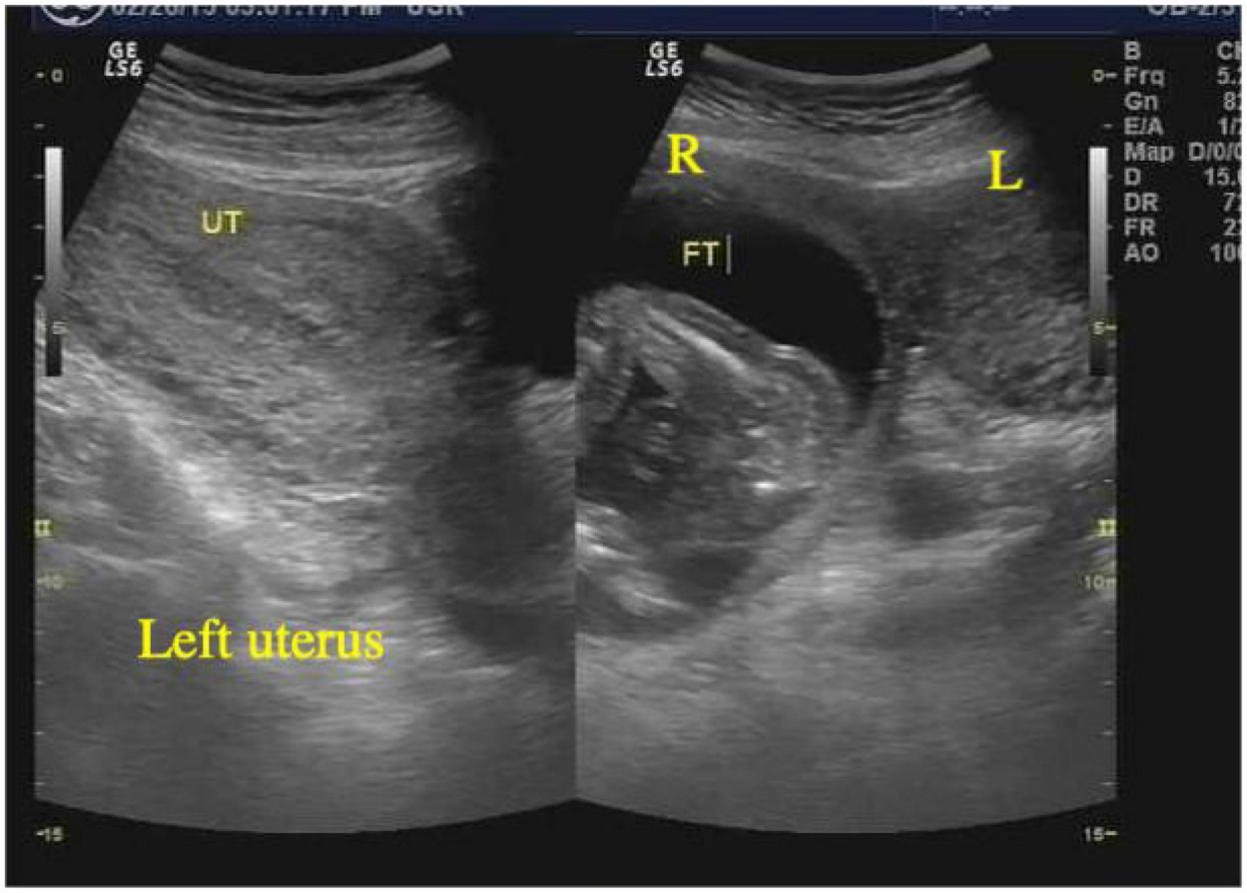
(1) The normal uterus on the left side of the patient is shown on the left side of the picture. (2) The right image shows both uteri visible simultaneously.

**Figure 2:**
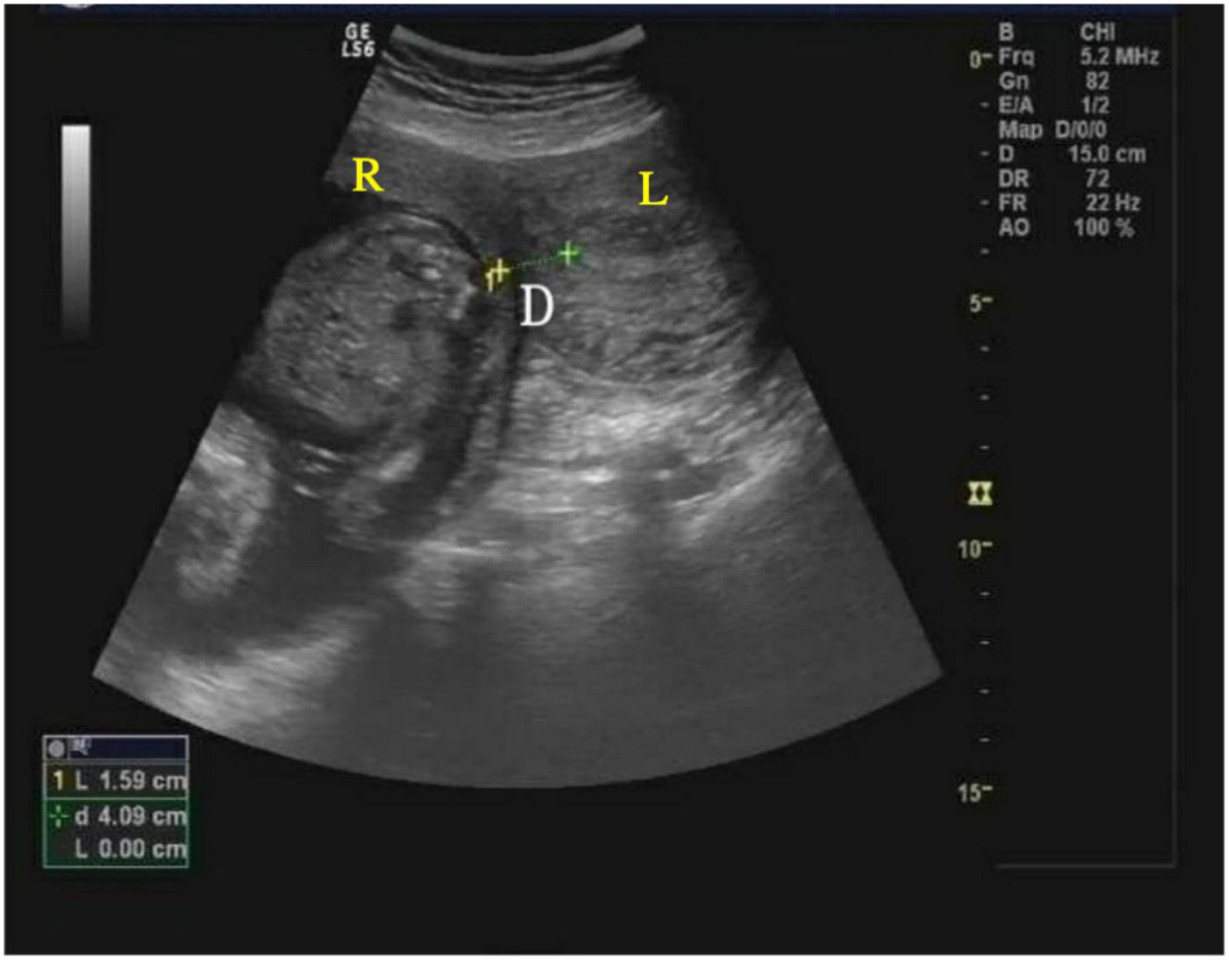
Distance between right residual uterine pregnancy and left non-pregnant uterine cavity (R represents the right residual horn uterus with pregnancy, L represents the left normal uterine body).

**Figure 3:**
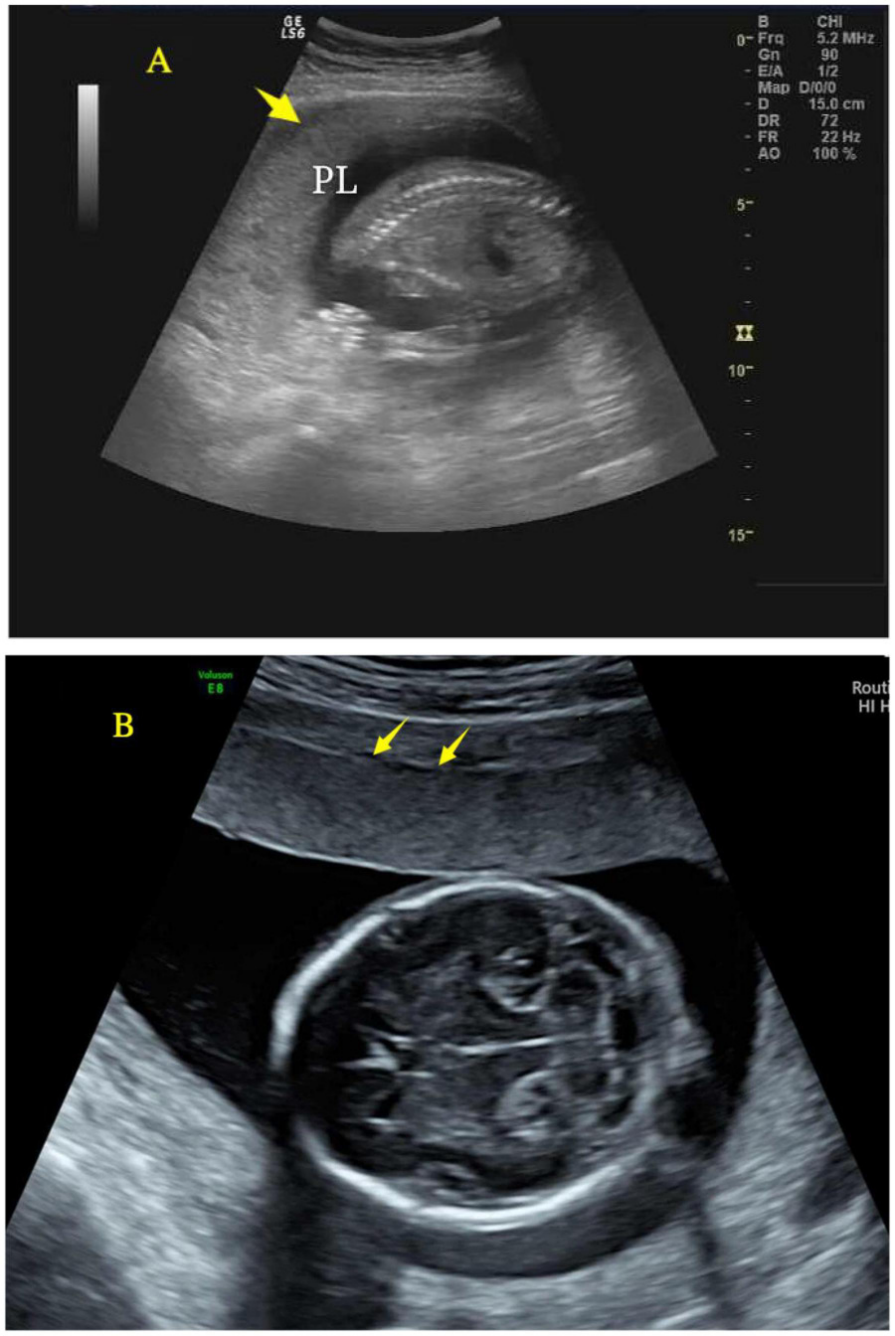
**(A)** This is an image of the placenta in our patient, with a blurred demarcation between the placenta and the myometrial wall shown by yellow arrows. **(B)** This picture shows a clear boundary between the placenta and the myometrial wall, as indicated by the double yellow arrows.

A 26-year-old primigravida at 20 weeks of gestation presented for a routine prenatal checkup without reporting any physical discomfort. Previous assessments had confirmed normal fetal development. The transabdominal ultrasound examination revealed an echogenic lesion in the left uterine corpus measuring 8.4 × 6.5 × 6.6 cm, with a homogeneous myometrium and an endometrial thickness of 1.5 cm, and adjacent to the right uterine wall, a gestational sac measuring 13.6 × 10.4 cm was detected (Figure 1). This sac was not connected to the left uterine cavity and was approximately 1.6 cm away from it (Figure 2). Fetal measurements revealed a biparietal diameter of 4.9 cm, head circumference of 17.5 cm, abdominal circumference of 15.5 cm, and femur length of 3.0 cm, with a fetal heart rate of 161 beats per minute. The fetal spine was aligned and continuous. Although the placenta was visible, it was poorly demarcated from the myometrium (Figure 3). Fetal heart activity and movement were observed, and the amniotic fluid depth was 5.7 cm. The ultrasound report indicated: ① A pregnancy adjacent to the right uterine wall, corresponding to 19 weeks and 6 days of gestation,the possibility of a rudimentary horn pregnancy or other types of ectopic pregnancies cannot be ruled out;② The potential for placenta accreta was also considered.

A few days after the ultrasound examination, the patient experienced severe abdominal pain, primarily localized to the lower abdomen, which was intolerable and accompanied by uterine contractions. Given the potential for serious complications, the clinical team conducted a thorough assessment of the patient's condition and recommended an emergency cesarean section. After careful consideration, the patient consented to the surgical intervention.

Intraoperative findings revealed a rudimentary uterus located on the right side, measuring approximately 9 × 7 cm. During manipulation of the right uterine corpus, spontaneous rupture of the myometrium occurred, leading to the release of clear amniotic fluid and the delivery of a stillborn male infant. The placenta was fully implanted within the rudimentary uterus. Surgical resection of the right rudimentary uterus and fallopian tube was performed, while the left fallopian tube and both ovaries were preserved. The patient's postoperative recovery was uneventful, and she was discharged on the fifth postoperative day. Histopathological examination confirmed the diagnosis of a rudimentary horn pregnancy.

## Discussion

Female genital anomalies are a group of congenital disorders caused by abnormal development of the paramesonephric ducts (i.e., Müllerian ducts) during the embryonic period. They include developmental abnormalities of the uterus, cervix, and vagina (5), and are often accompanied by anomalies in other systems, most commonly the kidneys and the skeletal system (6). Currently, various studies report that the prevalence of female reproductive organ malformations in the general population ranges from 0.5% to 6.7% (7). These patients typically have higher rates of miscarriage (25%), preterm birth (15%–25%), or cervical insufficiency (38%) (8). Other adverse outcomes include fetal growth restriction, abnormal fetal presentation, placental abnormalities, and ectopic pregnancy (9). In a study of more than 300 uterine pregnancies associated with Müllerian duct anomalies, patients with a unicornuate uterus had the highest rates of uterine rupture (4.7%) and *in vitro* fertilization-embryo transfer (12.6%) (10).

**Figure 4:**
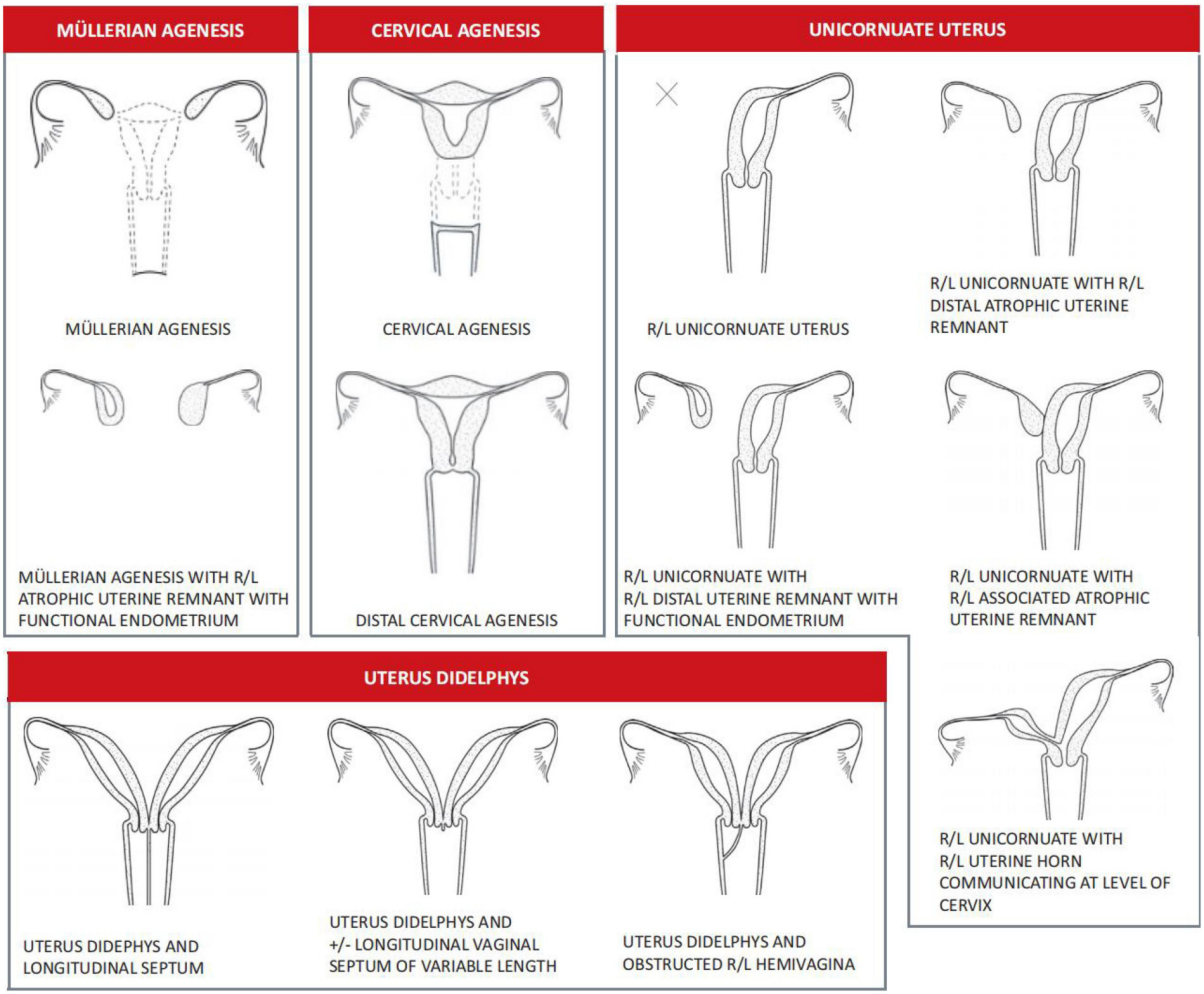
Classification of unicornuate uterus [American society for reproductive medicine (ASRM) müllerian anomalies classification 2021].

The unicornuate uterus, often accompanied by a rudimentary horn, results from incomplete development of one paramesonephric duct. It occurs in approximately 1 in 40,000 to 1 in 10,000 cases and is frequently associated with infertility and recurrent pregnancy loss, as the rudimentary horn presents an obstructive uterovaginal malformation (11). At the end of 2021, the ARSM Task Force created the 2021 ARSM Mueller Tube Development Abnormal Classification System (ARSM MAC 2021), they classified the unicorned uterus into five categories, including: (1) R/L unicornuate uterus, (2) R/L unicornuate with R/L distal atrophic uterine remnant, (3) R/L unicornuate with R/L distal uterine remnant with functional endometrium, (4) R/L unicornuate with R/L associated atrophic uterine remnant, (5) R/L unicornuate with R/L uterine horn communicating at level of cervix (Figure 4) (12).

During the ultrasound examination, we observed that the gestational sac containing the fetus in the rudimentary horn appeared to protrude outward at an angle from one side of the normal uterus. It lacked the typical morphology of the normal uterus, had a thinner myometrium, and was spatially separated from the normal uterine cavity. Although this presentation may resemble other types of ectopic pregnancies, fetuses in other ectopic pregnancies generally do not grow significantly because they often rupture early. It has been proposed that certain ultrasound findings (13), including a very thin perimyometrial layer, placental implantation, an empty uterine cavity, and a separate adnexal pregnancy sac, may aid in the diagnosis of rudimentary horn pregnancy. Our case exhibited several of these features. Unlike pregnancies in a bicornuate uterus, which are typically characterized by endometrial continuity between the pregnancy sac and the contralateral uterine horn, our case demonstrated discontinuity between the pregnancy sac and the normal uterine cavity.

Previous reports have indicated that 80%–90% of rudimentary horn pregnancies (RHP) result in horn rupture during the second trimester (14). Only 14% of cases are diagnosed before clinical symptoms appear. Most cases of RHP are challenging to diagnose and are often identified after rupture, which can lead to emergency surgery, blood transfusions, and increased morbidity. In our case, the patient had not been previously examined in our hospital, which caused great difficulty in our diagnosis, but due to the abnormal position of her gestational sac and the presence of a unicornuate uterus on the left side, our doctors suspected the possibility of a stumpy uterine pregnancy, but could not completely rule out other ectopic pregnancies, and most of the previously reported cases in the literature were detected in the early stages of pregnancy, with very few being detected in mid- to late-stage. And it is easy to misdiagnose as normal intrauterine pregnancy.

In the majority of case reports, patients either presented with clinical symptoms or had a known history of uterine anomalies (14). After a comprehensive review of the literature, we found that there is currently no unified standard for the extent to which ultrasound can diagnose the deformity of a unicornuate uterus. According to our clinical experience, with the more comprehensive knowledge of uterine malformations by current physicians and the development of ultrasound technology, unicornuate uterine malformations are generally easier to diagnose before pregnancy, and are not easily distinguishable when the morphology of the uterine cavity changes after pregnancy. It has been reported that only 22% of gynecologic cases and 29% of obstetric cases can be accurately diagnosed before surgery, indicating that the majority of cases can only be clearly diagnosed after surgery (15).

Pregnancy within a rudimentary horn is a rare form of ectopic pregnancy. It is caused by insufficient development of the myometrium, thinning of the decidual layer, and abnormal invasion of chorionic villi into the submembranous membrane, which leads to placental implantation. These conditions predispose the pregnancy to hemorrhage and uterine rupture, particularly in the second trimester. In rare cases, however, such pregnancies may continue to term. The risk of rupture in a non-communicating rudimentary horn depends on the degree of muscular development and hypertrophy, with rupture occurring between 5 and 35 weeks of gestation (16). The potential for rupture increases significantly after 16–20 weeks of gestation, presenting with symptoms similar to those of tubal pregnancy, such as abdominal pain and bleeding. Park et al. (17) reported that the risk of rupture can reach up to 50% in the second trimester. A thicker myometrium of the rudimentary horn compared to that of the fallopian tube may delay rupture, but when it occurs, the outcome can be life-threatening. Immediate surgical intervention is essential to prevent severe internal hemorrhage. In this regard, Jayasinghe et al. (18) recommend preemptive surgical removal of a rudimentary horn to prevent complications, such as dysmenorrhea and endometriosis, resulting from retrograde menstruation. This intervention is particularly important for patients with symptomatic non-communicating rudimentary horns, even before pregnancy occurs.

In reports on mid- and late-term pregnancies in women with uterine malformations, most cases required emergency surgical intervention based on the severity of the condition (19); however, other similar reports describe successful conservative management (20)^,^ with these cases primarily involving patients in the early stages of pregnancy. Preoperative rupture is one of the most serious complications of pregnancy in a rudimentary horn of the uterus, potentially leading to shock and life-threatening conditions (21). If a diagnosis of rudimentary horn pregnancy (RHP) is confirmed, surgical resection of the rudimentary horn is recommended due to the high risk of rupture during the second trimester (14). For patients with stable conditions or asymptomatic patients who refuse surgery, conservative treatment with methotrexate (MTX) may be considered, or surgical intervention may be performed based on the clinical presentation. Surgery serves both diagnostic and therapeutic purposes. Surgical options include radical resection of the rudimentary horn and the ipsilateral fallopian tube, or uterine-preserving evacuation of the gestational tissue. For non-communicating cases, the combination of MTX and laparoscopic surgery can reduce intraoperative blood loss (22).

If abnormal uterine development is detected during pregnancy, the specific type of malformation should be promptly identified, and its impact on both maternal and fetal outcomes should be assessed, with appropriate increases in the frequency of prenatal monitoring. In the event of premature rupture of membranes or signs of preterm labor, antibiotics and tocolytics should be judiciously administered under close surveillance to prolong gestation as much as possible (23). The timing and mode of delivery should be individualized to minimize complications for both mother and neonate. Although Müllerian duct anomalies are not an absolute indication for cesarean delivery, operative delivery should be considered in cases of malpresentation, fetal growth restriction (FGR), or a history of adverse pregnancy outcomes. Additionally, close attention must be paid to postpartum complications, including retained placenta and postpartum hemorrhage, to ensure maternal and neonatal safety (10).

Rudimentary horn pregnancy is an exceedingly rare condition that often presents without early, distinctive symptoms, making it highly susceptible to oversight and misdiagnosis. Consequently, the diagnosis of this condition predominantly relies on imaging diagnostics. Siwatch et al. (24) considered ultrasound as the most crucial diagnostic tool for identifying rudimentary horn pregnancies and emphasized the need for detailed ultrasound scanning to assess uterine anatomy and accurately locate the pregnancy, facilitating timely diagnosis and management. A report by Julio Elito Júnior et al. (25) described a heterotopic twin pregnancy, with one fetus developing within the cavity of a unicornuate uterus and the other in a non-communicating rudimentary horn. In that case, MRI was performed to confirm the diagnosis, while ultrasound was employed to monitor myometrial thickness and guide delivery planning, resulting in both fetuses surviving. The morphological changes of the uterus during pregnancy pose significant challenges for the diagnosis of uterine anomalies. As gestational age increases, the field of view in ultrasound examinations can be easily affected; therefore, women planning pregnancy should undergo relevant examinations beforehand to promptly detect uterine anomalies that may threaten maternal safety during pregnancy. For patients in early pregnancy, when an intrauterine gestational sac is not identified and a suspected adnexal mass is detected adjacent to the uterus, special attention should be paid to observing whether the uterine cavity morphology is abnormal, the relative position of the gestational sac to the uterus and endometrial cavity, and whether the gestational sac communicates with the cervical canal. When two-dimensional ultrasound is insufficient for diagnosis, three-dimensional ultrasound or MRI may be considered for further evaluation. These implications can directly inform clinical practice and guide the development of more effective treatment strategies.

## Data Availability

The original contributions presented in the study are included in the article/Supplementary Material, further inquiries can be directed to the corresponding author.

## References

[1] Cheng C Tang W Zhang L Luo M Huang M Wu X. Unruptured pregnancy in a noncommunicating rudimentary horn at 37 weeks with a live fetus: a case report. J Biomed Res. (2015) 29(1):83–6. 10.7555/JBR.29.20130089 25745480 PMC4342440

[2] Kadan Y Romano S. Rudimentary horn pregnancy diagnosed by ultrasound and treated by laparoscopy–A case report and review of the literature. J Minim Invasive Gynecol. (2008) 15(5):527–30. 10.1016/j.jmig.2008.05.010 18619925

[3] Ghotra MK Joshi B Bhutani S. Ruptured rudimentary horn pregnancy: delayed diagnosis. Cureus. (2021) 13:e15873. 10.7759/cureus.15873 34327098 PMC8302455

[4] Ural SH Artal R. Third-trimester rudimentary horn pregnancy. A case report. J Reprod Med. (1998) 43(10):919–21.PMID: 9800679 9800679

[5] Chinese Society of Obstetrics and Gynecology, Chinese Medical Association, Female Genital Anomalies Study Group, Chinese Obstetricians and Gynecologists Association. Chinese Experts' Consensus on the nomenclature and definition revision of female genital malformations (2022). Chin J Obstet Gynecol. (2022) 57(08):575–80. 10.3760cma.j.cn112141-20220321-00177 10.3760/cma.j.cn112141-20220321-00177 36008283

[6] Su S Bao XM Wang S Chen N Liu ZF Sun DW. Concomitant extragenital malformations of female reproductive tract anomalies: analysis of 444 cases in Peking Union Medical College Hospital. Zhonghua Fu Chan Ke Za Zhi. (2024) 59(5):346–52. Chinese. 10.3760/cma.j.cn112141-20231008-00136 PMID: 38797563 38797563

[7] Passos IMPE Britto RL. Diagnosis and treatment of müllerian malformations. Taiwan J Obstet Gynecol. (2020) 59(2):183–8. 10.1016/j.tjog.2020.01.003 32127135

[8] Parmar M Tomar S. Bicornuate uterus: infertility treatment and pregnancy continuation without cerclage: an unusual case. Open J Obstet Gynecol. (2014) 4:981–5. 10.4236/ojog.2014.415138

[9] Madhavi D. Bicornuate uterus-A case report. Anat Physiol Curr Res. (2012) 2:109. 10.4172/2161-0940.1000109

[10] Wang S Wang K Hu Q Liao H Wang X Yu H. Perinatal outcomes of women with müllerian anomalies. Arch Gynecol Obstet. (2023) 307(4):1209–16. 10.1007/s00404-022-06557-6 35426514 PMC10023634

[11] American College of Obstetricians and Gynecologists' Committee on Adolescent Health Care in collaboration with committee members Anne-Marie E. Amies Oelschlager A-ME, Berger-Chen SW. Management of acute obstructive uterovaginal anomalies: aCOG committee opinion, number 779. Obstet Gynecol. (2019) 133(6):e363–71. 10.1097/AOG.0000000000003281 31135762

[12] Pfeifer SM Attaran M Goldstein J Lindheim SR Petrozza JC Rackow BW. ASRM Müllerian anomalies classification 2021. Fertil Steril. (2021) 116(5):1238–52. 10.1016/j.fertnstert.2021.09.025. Erratum in: *Fertil Steril*. (2023) 119(6):1088. doi: 10.1016/j.fertnstert.2023.04.001. PMID: 34756327 34756327

[13] Blancafort C Graupera B Pascual MÀ Hereter L Browne JL Cusidó MT. Diagnosis and laparoscopic management of a rudimentary horn pregnancy: role of three-dimensional ultrasound. J Clin Ultrasound. (2017) 45(2):112–5. 10.1002/jcu.22393 27612443

[14] Li X Peng P Liu X Chen W Yang J Bian X. The pregnancy outcomes of patients with rudimentary uterine horn: a 30-year experience. PLoS One. (2019) 14(1):e0210788. 10.1371/journal.pone.0210788 30682068 PMC6347212

[15] Ağaçayak E Peker N Yavuz M Fındık FM Evsen MS Gül T. Rudimentary horn pregnancy—ten years of experience. Ginekol Pol. (2020) 91(3):117–22. 10.5603/GP.2020.0027 32266951

[16] Mehdizadeh K A Sadegi K Forghani F. Pregnancy in non-communicating rudimentary horn of A unicornuate uterus. Int J Fertil Steril. (2018) 11(4):318–20. 10.22074/ijfs.2018.5022 29043710 PMC5641466

[17] Park KJ Dominguez CE. Combined medical and surgical management of rudimentary uterine horn pregnancy. JSLS. (2007) 11:119–22. PMC3015782. PMID: 17651572 17651572 PMC3015782

[18] Jayasinghe Y Rane A Stalewski H Grover S. The presentation and early diagnosis of the rudimentary uterine horn. Obstet Gynecol. (2005) 105(6):1456–67. 10.1097/01.AOG.0000161321.94364.56 15932844

[19] Housni I Tanjona RA Rakotonirina MA Romuald R Fleurian S Halissiot BA. Uterine malformations and pregnancy: about 11 cases seen university hospital center of gynecology- obstetric befelatanana Antananarivo Madagascar. Int J Reprod Contracept Obstet Gynecol. (2020) 9(11):4670–6. 10.18203/2320-1770.ijrcog20204831

[20] Harpey AM McNierney BP O'Connell AA Lingo EG Wood EG. Ectopic pregnancy in a non-communicating rudimentary uterine horn: a case of successful medical management and literature review. Cureus. (2024) 16(10):e71856. 10.7759/cureus.71856 39559660 PMC11572001

[21] Hussain A Jawaid H Faisal N Shah N Kamal NS. Ruptured rudimentary horn pregnancy revealed on emergency laparotomy: a case of primigravida presenting in a developing country. Cureus. (2018) 10(5):e2591. 10.7759/cureus.2591 31489271 PMC6710493

[22] Ueda M Ota K Takahashi T Suzuki S Suzuki D Kyozuka H. Successful pregnancy and term delivery after treatment of unicornuate uterus with non-communicating rudimentary horn pregnancy with local methotrexate injection followed by laparoscopic resection: a case report and literature review. BMC Pregnancy Childbirth. (2021) 21(1):715. 10.1186/s12884-021-04195-5 34702216 PMC8547051

[23] Panagiotopoulos M Tseke P Michala L. Obstetric complications in women with congenital uterine anomalies according to the 2013 European society of human reproduction and embryology and the European society for gynaecological endoscopy classification: a systematic review and meta-analysis. Obstet Gynecol. (2022) 139(1):138–48. 10.1097/AOG.0000000000004627 34856567

[24] Siwatch S Mehra R Pandher DK Huria A. Rudimentary horn pregnancy: a 10-year experience and review of literature. Arch Gynecol Obstet. (2013) 287(4):687–95. 10.1007/s00404-012-2625-7 23183713

[25] Elito Júnior J Goldman SM Castro PT Werner H Sanchez VHS Araujo Júnior E. Heterotopic twin pregnancy in unicornuate uterus and non-communicating rudimentary horn with survival of both fetuses: magnetic resonance imaging and 3D reconstructions findings. J Clin Ultrasound. (2024) 52(8):1193–7. 10.1002/jcu.23747 38842403

